# The Grasp Strategy of a Robot Passer Influences Performance and Quality of the Robot-Human Object Handover

**DOI:** 10.3389/frobt.2020.542406

**Published:** 2020-10-19

**Authors:** Valerio Ortenzi, Francesca Cini, Tommaso Pardi, Naresh Marturi, Rustam Stolkin, Peter Corke, Marco Controzzi

**Affiliations:** ^1^Extreme Robotics Laboratory, School of Metallurgy and Materials, University of Birmingham, Birmingham, United Kingdom; ^2^The BioRobotics Institute, Scuola Superiore Sant'Anna, Pisa, Italy; ^3^Department of Excellence in Robotics and Artificial Intelligence (AI), Scuola Superiore Sant'Anna, Pisa, Italy; ^4^Australian Research Council (ARC) Centre of Excellence for Robotic Vision, Queensland University of Technology, Brisbane, QLD, Australia

**Keywords:** human-robot interaction (HRI), human-robot collaboration (HRC), seamless interaction, task-oriented grasping, object handover

## Abstract

Task-aware robotic grasping is critical if robots are to successfully cooperate with humans. The choice of a grasp is multi-faceted; however, the task to perform primes this choice in terms of hand shaping and placement on the object. This grasping strategy is particularly important for a robot companion, as it can potentially hinder the success of the collaboration with humans. In this work, we investigate how different grasping strategies of a robot passer influence the performance and the perceptions of the interaction of a human receiver. Our findings suggest that a grasping strategy that accounts for the subsequent task of the receiver improves substantially the performance of the human receiver in executing the subsequent task. The time to complete the task is reduced by eliminating the need of a post-handover re-adjustment of the object. Furthermore, the human perceptions of the interaction improve when a task-oriented grasping strategy is adopted. The influence of the robotic grasp strategy increases as the constraints induced by the object's affordances become more restrictive. The results of this work can benefit the wider robotics community, with application ranging from industrial to household human-robot interaction for cooperative and collaborative object manipulation.

## 1. Introduction

Traditional factories have already seen a progressive introduction of robots in very structured production lines. Such environments are designed to allow complete repeatability of tasks, which is very beneficial to the deployment of traditional robots (Billard and Kragic, 2019). However, robots must face different challenges in the context of the new wave of industrialization, i.e., the Fourth Industrial Revolution (Industry 4.0). The design principles of Industry 4.0 are said to be inter-operability, information transparency, technical assistance, and decentralized decisions (Østergaard, 2017). From this perspective, robots are envisioned to share their working space and actively cooperate with human workers taking into account their needs. Furthermore, robots are increasing their presence in several environments which are traditionally conceived to be completely human-centered, e.g., houses and hospitals. Object handover is a very common joint action performed multiple times in many cooperative scenarios. For example, when a human worker needs a screwdriver in a manufacturing plant, a robot might assist by fetching and handing the screwdriver to the worker. Similarly, in a domestic environment, a robotic helper might be asked to pass a wooden ladle or a glass bowl to the human chef.

### 1.1. Related Work

The handover action involves two agents who share the responsibility for the stability of the object, even if their goal differs (Mason and MacKenzie, 2005). The passer has to transport and present the object, while the receiver grasps it and uses it to accomplish a subsequent task. This collaborative interaction requires sensory feedback, mutual understanding, and coordination between the two agents, who exchange signals and cues to adjust their behavior (Basili et al., 2009; Ramenzoni et al., 2011; Endo et al., 2012; Strabala et al., 2012; Controzzi et al., 2018). Several aspects of the object handover have been investigated by the robotic research community to better understand these mechanisms and improve human-robot collaboration. For instance, previous studies examined trajectory and velocity of the agents' arm approaching movement (Huber et al., 2008a,b; Prada et al., 2013; Parastegari et al., 2017) and the control of the passer's grasping force on the object during the handover (Mason and MacKenzie, 2005; Chan et al., 2013; Parastegari et al., 2016, 2018; Controzzi et al., 2018). Three fundamental aspects for an efficient, comfortable and intelligible handover are: (1) the location where the object is transferred; (2) the orientation of the object toward the receiver; and (3) which part of the object is offered unobstructed (Cakmak et al., 2011a; Cini et al., 2019). Previous studies suggested that it is the passer who mainly selects the handover location (Shibata et al., 1995) and can use subtle cues, such as shared gaze, to dictate it (Moon et al., 2014). The choice of the handover location is affected by several factors, such as physical characteristics of the partners (Parastegari et al., 2017; Kato et al., 2019), and their distance and urgency to exchange the object (Mainprice et al., 2012). Similarly, it is fundamental that the passer presents the object with an appropriate configuration, thus deciding both its orientation and which part of the object to offer unobstructed to the receiver. Cakmak et al. (2011b) compared handover configurations learned from human examples vs. configurations planned using a kinematic model of the human body, and reported that the learned configurations were preferred in terms of usability, appropriateness, and naturalness even if planned configurations provided better reachability. In addition, the same authors (Cakmak et al., 2011a) hypothesized that object tilt (i.e., orientation) and obstruction may help to communicate the intention of handing over, but they did not find any influence of the passer's grasping strategy on the delay of the receiver in taking the object from the robot. Occlusion and orientation seem to gain even more importance when objects have clear affordances (i.e., object parts that suggest specific actions, such as handles for grasping Gibson, 1977; Chemero, 2003; Montesano et al., 2008; Osiurak et al., 2010). In an observational study, Strabala et al. (2013) observed that passers were inclined to grasp and rotate the object in order to facilitate the receiver to grab the object's affordance. Aleotti et al. (2014) developed a robotic system that was able to deliver objects orienting their affordances (defined in advanced for each object) toward the receiver. With a preliminary user study, they showed that their solution improved sense of comfort and safety, decreasing the reaction time of the receivers, with respect to a system that disregards object orientation. Recently, Chan et al. (2019) proposed a method that enables robots to automatically recognize object affordances and to choose the proper handover orientation starting from the observation of the usage and handover of a set of objects. Nonetheless, no user study was performed to test this system. All these previous studies investigated the effects of different grasp strategies of the passers on the handover action, but their protocol did not consider a following task for the receiver. A handover is usually performed to help the receiver to accomplish a subsequent operation. Thereby, a protocol must include a subsequent task to aim at evaluating the quality of the human-robot collaboration (Ortenzi et al., 2019). Our previous study on humans (Cini et al., 2019) suggested that during a handover, human passers adjust both their grasp type and location to better accommodate the receiver's needs. While power grasps, that are stable and enveloping, are favored for a direct use of the object, passers preferred precision grasps leaving most of the object available during a handover. Moreover, passers are careful to not obstruct the object affordances particularly when the receiver needs to use them to accomplish the following task. These findings not only confirm that humans adjust their grasp according to their own task (such as passing an object), but it underlines that the passer's grasping strategy is affected also by the subsequent task of the partner. However, the effects of such adjustments on the efficiency and on the perception of the receiver during a realistic interaction (i.e., where two actors hand over an object with the purpose of using it, and not only of exchanging it) has not been assessed yet.

### 1.2. Contributions

This work aims to investigate how the grasping strategy of a robotic passer affects: (i) the reaching time of human receivers toward the handover location; (ii) the performance of human receivers during the execution of a subsequent task; (iii) the compensatory manipulations performed after the handover; and (iv) the receivers' perception of the collaboration. In our experiment, twenty-two participants with no prior experience with robots were asked to grasp five test-objects from a robot and to perform a specific subsequent task with each object. The object list included a drill, a mug, a mustard bottle, scissors and a screwdriver. Each object was presented individually by the robot in two different conditions: an Unaware Grasp (UG condition) or a Task-oriented Grasp (TG condition). The conditions differ in hand placement and object occlusion. The grasps used in UG were selected using a well-known robotic grasping algorithm, SIMOX (Vahrenkamp et al., 2013), based on stability considerations. Differently, grasps used in TG were hand-picked based on the results of (Cini et al., 2019) and considering the task to perform after the handover. Each condition was repeated three times (three trials), and for each object the two conditions were performed sequentially (for a total of six trials per object). We compared the duration of receiver's arm movements toward the robot and the completion time of the receiver's subsequent task for each object across the conditions, to measure how the grasp strategy of the robot passer impacts the approach and the performances of the receiver. We analyzed the video recordings frame by frame and manually classified the manipulative re-adjustments of the objects performed by receivers in each condition soon after the handover. We discriminated between three classes: no-adjustment, in-hand adjustment, or bi-manual adjustment (Yousef et al., 2011; Visser et al., 2014). Finally, we administered a questionnaire to th e subjects to obtain subjective metrics describing perceptions during the collaboration. The ratings of the two experimental conditions were compared for each object. Our results show that the different conditions do not alter the duration of the receivers' approaching movement; however, they strongly affect the receiver's performances of the subsequent task and the perceptions of the interaction. We found a significant decrease of the task completion time and of the number of object re-manipulations in TG with respect to UG. In addition, participants expressed a clear preference for TG, judging the robot's behavior more collaborative and the task performance in general faster and easier in TG than in UG. The effect of the passer's grasping strategy resulted especially noticeable for those classes of objects whose affordances and use introduce strong constraints in the manipulative actions of the receiver.

## 2. Materials and Methods

### 2.1. Participants

22 subjects (gender: 12 male, 10 female; age: μ = 35.1, σ = 9.5) took part in the experiment. All participants were healthy, reported normal vision, and were not aware of the purpose of the experiment. Twenty-one (out of 22) participants were right-handed and used their right hand. One participant was ambidextrous and used his right hand. All participants had no (or negligible) experience with robots and robotics. This ensured that their background or experience did not help during the interaction with the robot (biasing the measure of our objective and subjective metrics). All subjects participated on a voluntary basis and gave their signed consent to the participation. Each participant took 60–80 min to complete the experiment. This project has full ethical approval from the Science, Technology, Engineering and Mathematics Ethical Review Committee at the University of Birmingham, UK (Application for Ethical Review ERN_19-0671).

### 2.2. Experimental Setup

**Figure 1:**
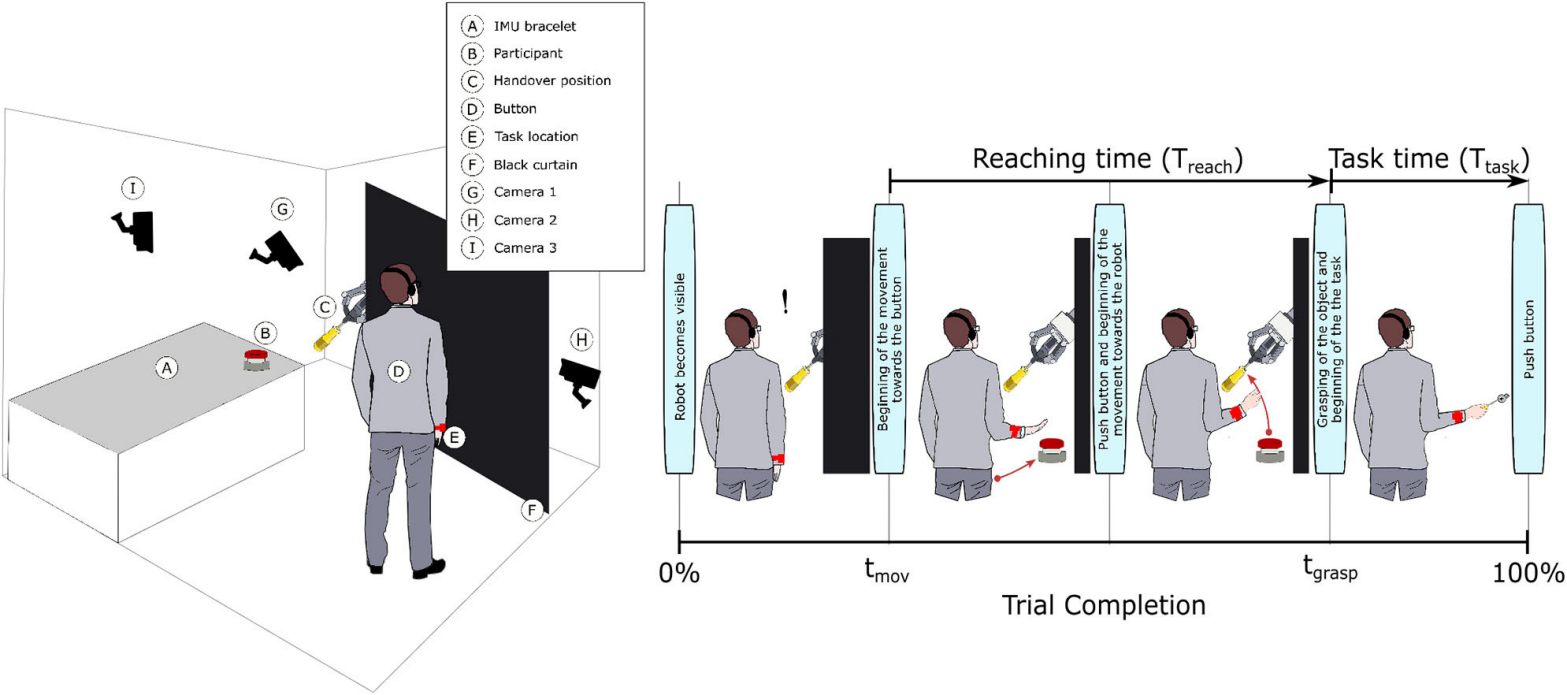
Experimental setup and protocol. The left-hand side picture shows the experimental setup, with handover position, cameras' location and participants' starting point. The right-hand side picture shows a trial with the screwdriver in TG.

In this study, we used a KUKA iiwa (7 degrees of freedom) manipulator equipped with a Schunk SDH 3-finger hand and a force/torque sensor (sampling rate of 2KHz) mounted on the wrist. The robot handed over 5 test-objects chosen from the YCB dataset (Calli et al., 2015a,b, 2017): a mustard bottle, a screwdriver, 3D-printed scissors, a 3D-printed mug, and a 3D-printed drill. Tasks were performed on a table. The setup included also a button, connected to a timer and placed on the same table, and three RGB commercial cameras (Logitech 4K, at 30 fps) recording the workspace from different points of view, as shown in Figure 1. The supplementary objects used in the tasks consisted of a plastic jug, cylinders made of plasticine, an arrow signal mounted on a rigid frame and a box. Mustard bottle (test-object) and plastic jug (supplemental object) were filled with a measured amount of rice. Each object had a fixed designated area (repeatable) on the table. An IMU (LPMS-URS2: 9-axis and connected via USB) was used to record the movements of the participant's right hand at 100 Hz. The IMU was placed on the wrist of the participant's right arm by means of a custom-made bracelet. A black curtain was used to hide the home position of the robot.

### 2.3. Experimental Protocol

In this experiment, participants were asked to play the role of receiver and to collaborate with a robotic passer. Subjects had to receive 5 test-objects from the robot using their right hand and subsequently use the grasped object to perform a task in the shortest possible time. The experiment comprised of two conditions that differed in the grasping strategy used by the robot to present the object (Table 1). In condition UG, the robot's grasping strategy was based only on the stability of the grasp and accounted for neither the handover action nor the following task that the receiver had to perform with the object. We used SIMOX (Vahrenkamp et al., 2013) to plan the grasps autonomously. We used the model of the Schunk Dexterous Hand 2 already in SIMOX and the model of the objects from the YCB object dataset website. We hand-picked two configurations for the Shunk hand that were exemplary of a centric grasp and a cylindric grasp. In the centric grasp we hand-picked, the degree of freedom at the base of the two fingers is rotated 60°; instead, in the selected cylindric grasp, the same joint was set to 0 (i.e., the first joints of each of the two fingers were parallel). We simulated 50 grasps per hand “configuration” per object (i.e., to explore the remaining possible joints configurations for the two type of grasps), for a total of 2 configurations × 50 simulations × 5 objects = 500 total grasps. For each grasp, we checked object occlusion and grasp quality measure (stability based on the Grasp Wrench Space Computation). All computed grasps had force closure. The majority of the grasps generated by the simulator covered at least a portion of the graspable functional part of the object (more than 66% for the mustard bottle and more than 73% for all the other objects). We then selected the grasp configuration with the higher grasp quality within this set of proposed simulated configurations. In condition TG, the robot's grasping strategy was selected following the findings in (Cini et al., 2019) and it ensured that the subjects had the possibility to easily grab the objects' grasping affordances and perform the following action right away. In other words, the chosen robotic grasps left the grasping affordances completely free for the receiver, and stably held the remaining body of the object to safely hand it over. In each condition, the objects were placed in the robot hand by the experimenters (avoiding the uncertainties induced by a sensorial feedback guiding an autonomous robotic grasping). Before the beginning of each trial, the robot was moved in its home position behind the curtain to hide the grasps from the participants (Figure 1), and the experimenters inserted the object in the pre-determined position inside the hand, which was software-driven to the predefined hand configuration. In the meantime, the participants were asked to stand in front of the curtain staring at a red dot placed on the curtain and keep their right hand (instrumented with the IMU) alongside their body with their feet aligned to marks on the floor. Participants were wearing headphones in order to not hear the robot's motor noise that would have signaled the start of the motion of the robot. The rationale behind this is that we did not want the participants to start the trial in anticipation to the robot becoming visible. The start of each trial was signaled by a beep sound through the headphones. After the beep sound, the software waited a random time (≤10 s), then the robot moved out of the curtain and reached the handover position where it waited for the receiver to grasp the object. The participants were instructed to maintain the starting position until they could see the robot. From the moment the robot became visible, they were allowed to move at any time and press the button, grasp the object from the robotic gripper, use it to perform a task, place the object back on the table in the appropriate position, and press the button again (signaling the end of the task, Figure 1 right). Subjects were instructed to perform all these actions as quickly as possible, grasping the object from the robot only with their right hand. However, they could perform in-hand and/or bi-manual manipulation of the grasped object, if needed, in order to accomplish the following task. Similarly to the SHAP test (Light et al., 2002), tasks were object-specific, including common daily life actions as inserting, pouring, cutting, screwing. Tasks were designed ensuring their repeatability among trials. More details on each object-specific task can be found in Table 1. In addition, the position of each additional object involved in the tasks as well as the final position of each test-object was marked on the table and was kept constant throughout the experiment. Likewise, the starting position of participants, the position of the curtain, the home position, and handover position of the robot were fixed. The object releasing strategy used by the robot was the following: whenever the force/torque sensor at the robot's wrist detected a contact (i.e., the absolute value of the force recorded by the sensor exceeded a fixed threshold), the gripper opened, releasing the object. The releasing strategy is out of the scope of this study. The experimental protocol foresaw that for each object, the two experimental conditions were performed sequentially, repeating each condition three consecutive times, for a total of six trials per object and a total of 22 subjects × 5 objects × 2 conditions × 3 repetitions = 660 trials over the entire experiment. The order of the conditions was randomized across participants to have half of participants performing UG as first condition for each object, and the other half having UG as the second condition. The order of the test-objects was also chosen randomly. Participants were only informed about the experimental protocol: before the beginning of the trials associated with each object, the experimenters showed the task to perform with the object, and informed the participants that they would experience two experimental conditions (each of them with three trials in a row). However, participants were not aware of the differences between the experimental conditions and which condition (UG or TG) they would have experienced first. At the end of the six trials for each object, participants were asked to rate the collaboration in the two conditions by means of a questionnaire based on a Likert scale of 7 points (one strongly disagree; seven strongly agree). The questionnaire included some of the metrics proposed in (Dragan et al., 2015; Hoffman, 2019) for evaluating human-robot collaboration, but some questions were slightly modified and adapted to our setup. The full questionnaire is reported in **Table 3**. Before starting the experiment, each participant performed a training session where the robot handed over a ball (abstract object). The training was <5 min long, and it had the purpose to let the participant familiarize with the experimental apparatus.

**Table 1:** Experimental conditions.

**Object**	**Experimental conditions**		
	**UG grasp**	**TG grasp**	**Task**
Mustard bottle	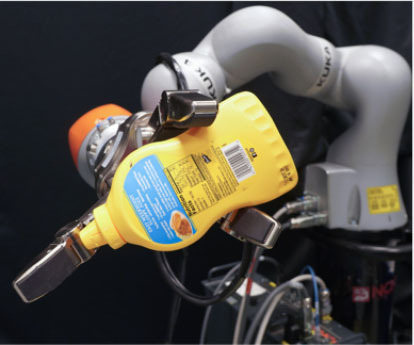	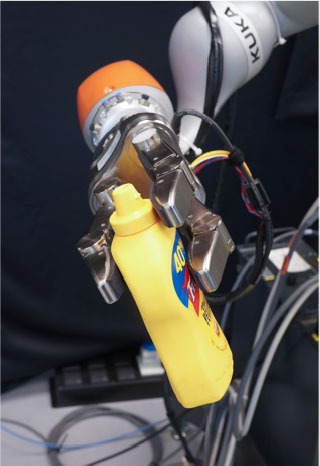	Press the button, receive the mustard bottle from the robot, open it (by unscrewing cap), and whilst ensuring as little spillage as possible, pour its contents into the bowl present on the table. Place the bottle on the table and then press the button again. Perform the task as quickly as possible.
Screwdriver	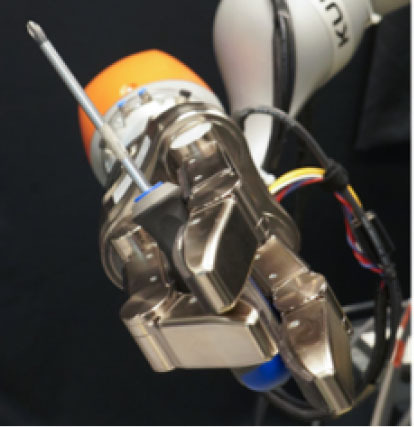	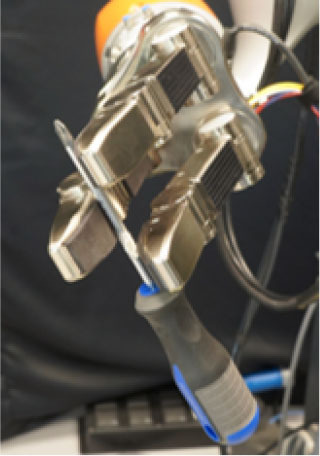	Press the button, receive the screwdriver from the robot and use it to rotate the screw half a turn clockwise, or beyond the red mark. Once completed, the screwdriver should be placed back on the table and press the button again. Only the right hand should be used for turning the screw. Your other hand can be used to steady the frame of the arrow unit. Perform the task as quickly as possible.
Drill	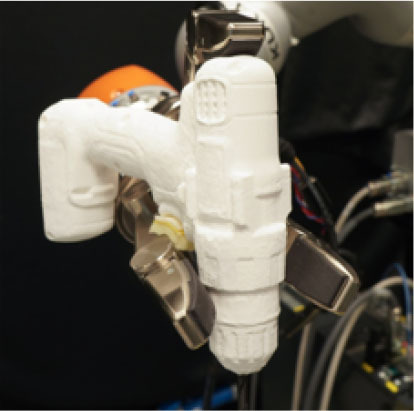	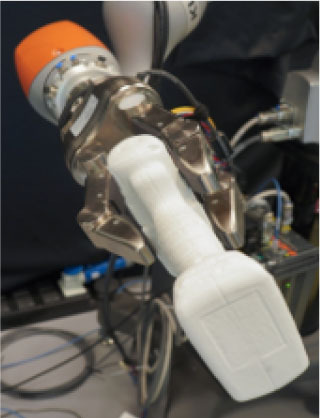	Press the button, receive the drill from the robot, push the tip of the drill into the hole (while pushing the drill's button). You can use the other hand to stabilize the plastic frame. Place the drill on the table in its labeled position and then press the button again. Perform the task as quickly as possible.
Mug	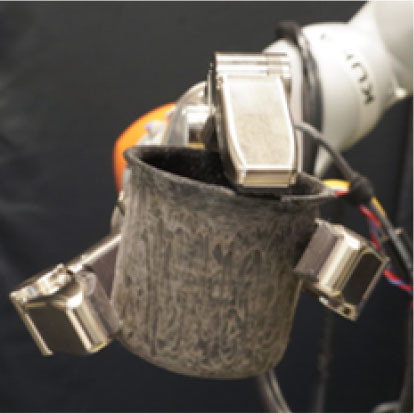	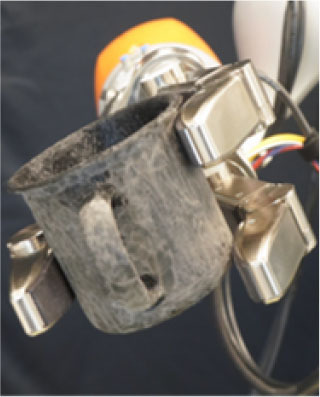	Press the button, receive the mug from the robot, pour the contents of the jug (placed on the table) into the mug, avoiding spilling. Place both mug and jar on the table in their labeled position and then press the button again. Perform the task as quickly as possible.
Scissors	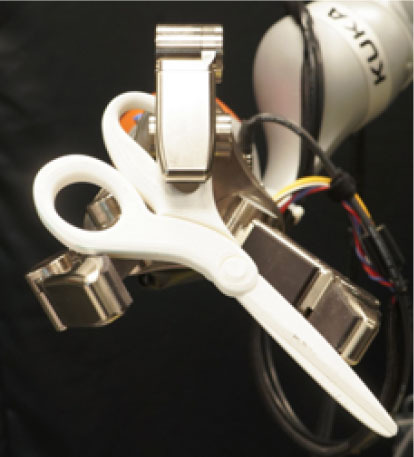	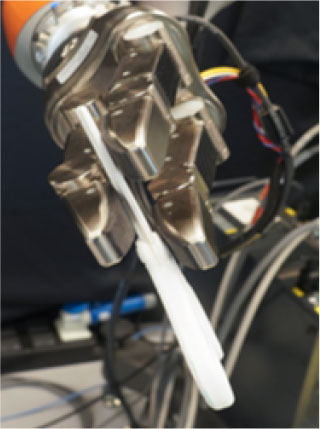	Press the button, receive the scissors from the robot, cut the cylinder of into two chunks. You can use the second hand to stabilize the plasticine. Place the scissors on the table in their labeled position and then press the button again. Perform the task as quickly as possible.

*Photos of the grasps in UG and TG conditions and a brief description of the task to perform are reported for each object*.

### 2.4. Data Analysis

**Figure 2:**
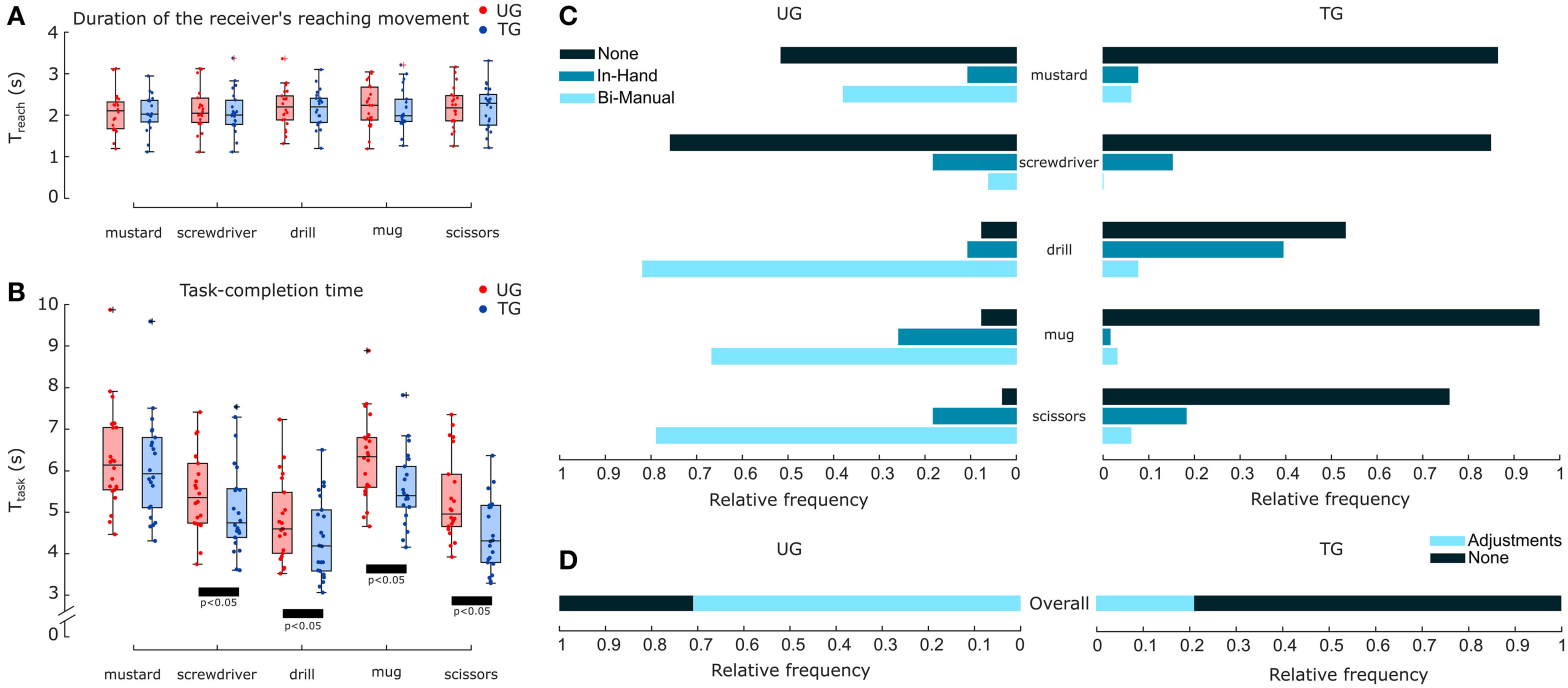
Duration of the reaching movement, of the task and comparison of the object manipulative adjustments between UG and TG for each object. **(A)** Distribution of the duration of the receiver's reaching movement (*T*_*reach*_) of all the 22 participants for each object and experimental condition. None of the comparisons performed on *T*_*reach*_ using the Wilcoxon test resulted significant (*p* > 0.5). **(B)** Distribution of the task-completion time (*T*_*task*_) of all the 22 participants for each object and experimental condition. Black bars represent significant comparisons (*p* < 0.05) performed on *T*_*task*_ using the Wilcoxon test. **(C)** Relative frequencies of object manipulations performed by the participants with each object across the two experimental conditions. The frequencies shown are normalized by the total of 66 object manipulations performed with each object in each condition. **(D)** Relative frequencies of the overall adjustments performed by the participants in each experimental condition. The class Adjustments includes both in-hand and bi-manual manipulations. The frequencies shown are normalized by the total of 330 object manipulations performed in each condition.

**Figure 3:**
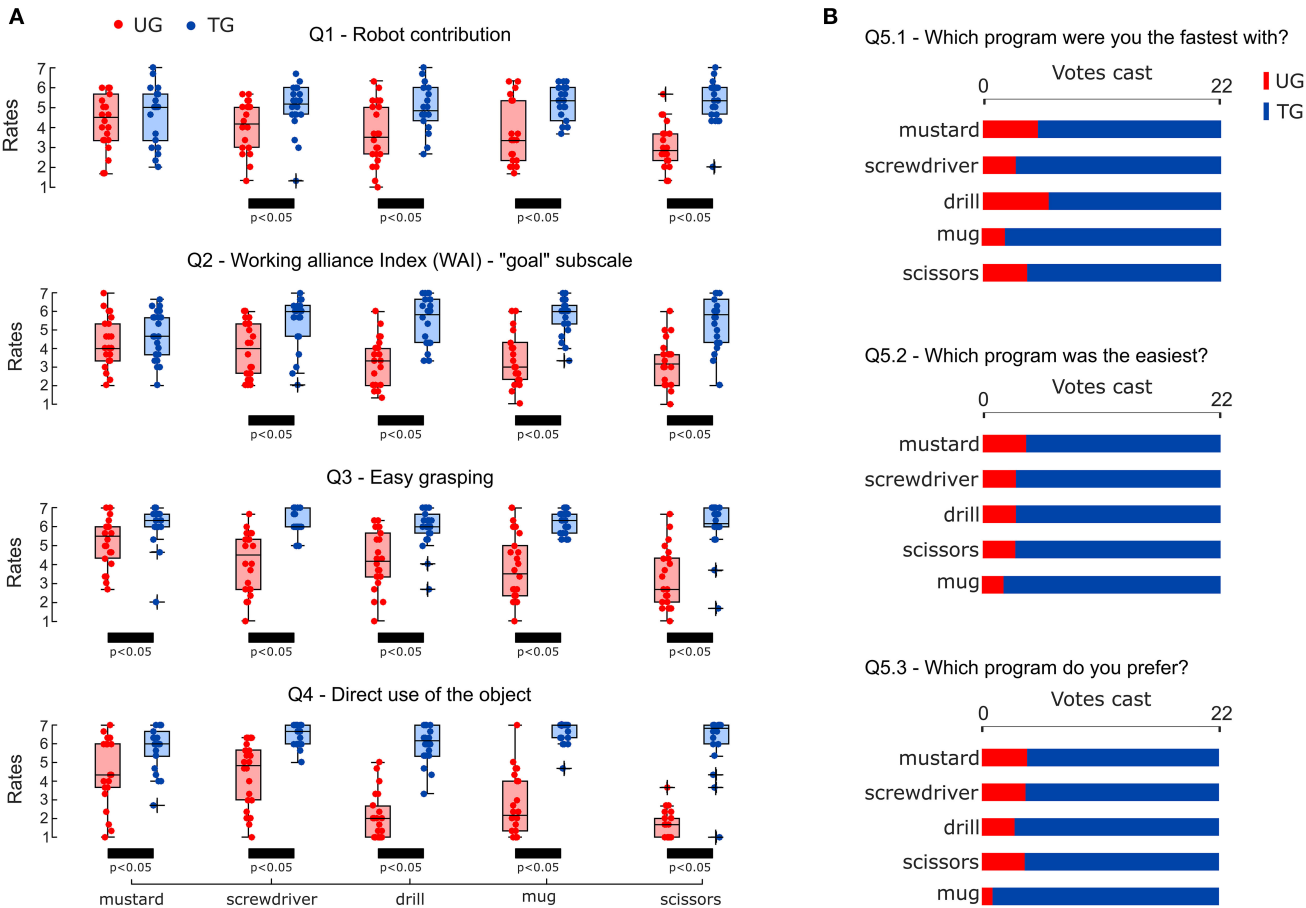
Questionnaire results. **(A)** Comparison of the mean scores gave by each of the 22 participants to each set in both conditions (UG and TG). Black bars represent significant comparisons (*p* < 0.05). **(B)** Results of the Forced choice questions.

We segmented the trials using the signals acquired from the force/torque sensor mounted on the robot's wrist, the IMU, and the button. We found the onset of the receiver's arm movement *t*_*mov*_, starting from the instant when the button was pressed the first time and moving backward along the acceleration profile of the receiver's arm until the signal dropped below 0.1 G. Then, in each trial we evaluated the time spent by the participant to execute the reaching movement and to execute the task. The duration of the reaching movement was evaluated as the time difference between the instant when they made contact with the object, *t*_*grasp*_ (obtained with a contact detection with the force/torque sensor readings triggering the release of the object by the robot), and *t*_*mov*_. The time spent to complete the task was evaluated as the absolute time difference between *t*_*grasp*_ and the instant when the participant pushed the button after completing the task. Then, we computed the mean of the duration of the reaching movement (*T*_*reach*_) and of the execution time (*T*_*task*_) over the three trials for each triplet (participant, condition, and object). We aimed to investigate whether the receiver's approaching movement or the execution time (and therefore the efficiency) of the task was affected by the grasping strategy used by the robot to present the object. To answer this question, for each object we compared *T*_*reach*_ and *T*_*task*_ across the two conditions (UG and TG) using the Wilcoxon test. Non-parametric Wilcoxon test was chosen since not all the groups of data resulted to be normally distributed and presented outliers and/or heavy tails (Figures 2A,B). We analyzed the videos recorded by the three RGB cameras in the experimental setup to investigate whether the participants needed to re-manipulate the object before using it for the subsequent task. In particular, we manually classified each trial of the experiment according to the type of object manipulation performed by the participants, thus distinguishing the trials in three classes: (i) no-adjustment, (ii) in-hand adjustment, and (iii) bi-manual adjustment. We classified as “no-adjustment” all those trials in which participants were able to grasp the object from the robot and use it straight away with no need of any further manipulations. We identified all adjustments performed by the participants with a single hand in order to change the position and/or orientation of the object as in-hand adjustments (Exner, 1992; Yousef et al., 2011; Visser et al., 2014). In-hand manipulations included the translation of the object from the fingertips to the palm (or vice versa), the shift of the object in a linear manner along or across the fingers (e.g., repositioning a pencil for writing), and rotation of the object. Finally, we labeled as bi-manual all those adjustments performed using both hands. Once the analysis of the videos was completed, we computed the relative frequencies of the occurrences of each adjustment type for each object and condition (UG and TG) of the experiment. In addition, we compared the total number of no-adjustment and of manipulative adjustments (evaluated as the sum of occurrences of in-hand and bimanual adjustments) performed in UG and TG. The internal consistency of each set of questions of the questionnaire was measured with Cronbach's alpha. Then, for all sets but the Forced-Choice questions, we carried out the following analysis. For each object and condition, we evaluated the score of each set (*S*_*m*_) computing the mean across the scores achieved by their internal items. To evaluate the influence of the robot's grasping strategy on different aspects of the human's perception of the task, a comparison for each object and for each set was performed between *S*_*m*_ in UG and TG using the Wilcoxon test, since not all the groups of data were normally distributed and presented outliers and heavy tails (Figure 3A). Finally, we analyzed the results of the Forced-Choice question annotating for each object how many participants preferred condition UG or TG. Statistical significance of all the tests performed in this work was defined for *p*-values < 0.05. In addition, the effect size of performed each Wilcoxon test, was evaluated using the correlation coefficient $r=\frac{Z}{\sqrt{(}N)}$, where *Z* is the *Z* score of the Wilcoxon statistics and *N* is the total number of the observations (equal to 44, 22 participants × 2 conditions) (Fritz et al., 2011; Tomczak and Tomczak, 2014). *r* can be interpreted using the Cohen's guidelines (Cohen, 1988) that consider values of 0.10, 0.30, and 0.50 to be indicative of small, medium, and large effects, respectively.

## 3. Experimental Results

All participants successfully performed the tasks in both conditions; however, their performances and their perceptions differed markedly.

### 3.1. Duration of the Receivers' Reaching Movement

To characterize the receiver's reaching movement, we evaluated its duration as the time elapsed from the onset of the receiver's arm movement toward the button until the contact with the object held by the robotic passer. For each object and participant, we evaluated the mean value of the reaching movement duration *T*_*reach*_ over the three repetitions of each condition (Figure 2A). The impact of the robot's grasping strategy on the receiver's approaching movement was investigated by comparing *T*_*reach*_ in conditions UG and in TG using the Wilcoxon test (Table 2). We found that *T*_*reach*_ did not differ across conditions for any object (*p*-values > 0.5).

**Table 2:** Statistical results.

		**Drill**	**Mug**	**Mustard**	**Scissors**	**Screwdriver**
*T*_*reach*_	*Z*	0.601	1.023	−0.016	0.438	1.575
	*p*	0.548	0.307	0.987	0.661	0.115
	*r*	0.091	0.154	0.003	0.066	0.237
*T*_*task*_	*Z*	−2.289	−3.036	−1.217	−3.360	−2.191
	*p*	0.022^*^	0.002^**^	0.223	0.001^**^	0.028^*^
	*r*	0.345	0.458	0.184	0.507	0.331
Robot contribution	*Z*	−3.249	−3.462	−1.451	−3.768	−2.910
	*p*	0.001^**^	<0.001^***^	0.146	<0.001^***^	0.004^**^
	*r*	0.490	0.522	0.219	0.568	0.439
Working Alliance Index (WAI)	*Z*	−3.728	−3.625	−1.166	−3.387	−3.274
	*p*	<0.001^***^	<0.001^***^	0.244	<0.001^***^	0.001^**^
	*r*	0.562	0.547	0.176	0.511	0.494
Easy grasping	*Z*	−3.125	−3.687	−2.582	−3.755	−3.725
	*p*	0.002^**^	<0.001^***^	0.01^**^	<0.001^***^	<0.001^***^
	*r*	0.471	0.556	0.390	0.566	0.562
Direct use of the object	*Z*	−3.929	−3.927	−2.216	−3.936	−3.948
	*p*	<0.001^***^	<0.001^***^	0.027^*^	<0.001^***^	<0.001^***^
	*r*	0.592	0.592	0.334	0.593	0.595

*Results of the comparison between conditions UG and TG performed on each object using Wilcoxon test. Statistical significance was defined for p-value < 0.05. The statistical significance is reported according to the following notation*:

* *p < 0.05*,

** p < 0.01, and

*** *p < 0.001 (All the p-values < 0.05 are bolded). The effect size of each test was evaluated using the correlation coefficient r = Z/sqrt(N), where N is the total number of observations on which the Z is based (N = 44, 22 participants × 2 experimental conditions) (Fritz et al., 2011). Small effect size is defined for r < 0.3, medium and large effect sizes are defined for values of 0.3 < r < 0.5 and r > 0.5, respectively (Cohen, 1988)*.

### 3.2. Task-Completion Time

Task performance was evaluated with the task-completion time measured as the time elapsed from the moment the participant made contact with the object (when held by the robotic passer) until the end of the task. For each object and participant, we evaluated the mean value of task-completion time *T*_*task*_ over the three repetitions of each condition (Figure 2B). The impact of the robot's grasping strategy on the task performance was investigated by comparing *T*_*task*_ in conditions UG and in TG using the Wilcoxon test (Table 2). We found that the task-completion time in TG was significantly lower than in UG for most of the objects. The only exception was with the mustard bottle, whose task-completion time did not statistically differ across conditions (*p*-value = 0.223 and effect size *r* = 0.184).

### 3.3. Object Manipulation

The task efficiency is negatively impacted by the compensatory manipulative actions the participants were compeled to perform to be able to comfortably execute the subsequent task. The manipulations were categorized in: (i) no-adjustment, (ii) in-hand adjustment, and (iii) bi-manual adjustment. In this work, a total of 660 trials were manually labeled according to the type of adjustment exploited by the participants: 66 per object and per condition (Figure 2C). UG reported a high number of bi-manual adjustments and displayed a relative frequency >60% of the occurrences with the drill, mug and scissors. In-hand adjustments in UG were limited with all the objects and their frequencies did not exceed the 26% of occurrences. No-adjustment was performed in more than 50% of cases only with mustard bottle and screwdriver, but in <10% of cases with drill, mug and scissors. In comparison, the number of occurrences of no-adjustment in TG sharply increased, reaching a frequency of 53% for the drill and above 75% for the other objects. This resulted in a significant decrease of in-hand and bi-manual adjustments. In condition TG, bi-manual adjustments were performed in <10% of cases for all the objects and the frequency of in-hand adjustments reached 39% of occurrences only for the drill, while it did not exceed 20% of cases for the other objects. Comparing the overall number of object manipulations performed in each condition (Figure 2D), the frequency of manipulative adjustments (including both in-hand and bi-manual adjustments) decreased from 71% in UG to 21% in TG.

### 3.4. Subjective Measures

The participants' perceptions of the interaction with the robot and their preference between the two conditions were assessed by means of a questionnaire. This questionnaire was administered after performing both conditions (UG and TG) for each object. The test consisted of five sets of three questions, each answered based on a Likert scale of 7 points (one strongly disagree; seven strongly agree) (Table 3). The internal consistency of each set was assessed using Cronbach's alpha. The results showed that the more general sets evaluating the robot contribution to the team and the working alliance between participants and robot (Q1 and Q2) had a good consistency (0.8 < α < 0.89), while the consistency of the other sets reached excellent scores (α > 0.899). Except for the Forced-choice questions (Q5), the score of each set (*S*_*m*_), defined as the mean across the scores of the questions, was evaluated for each object and for both experimental conditions (Figure 3A). In order to investigate the effect of the robot's grasping strategy on the human's perception of the task, for each object and for each set, a comparison of *S*_*m*_ in UG and in TG was performed using the Wilcoxon test. Results showed that only *S*_*m*_ obtained for the mustard bottle on the Robot contribution and Working alliance index sets (Q1 and Q2) did not significantly differ across condition UG and TG. Except for these two cases, all the scores achieved by the sets in TG were statistically higher than those achieved in UG (*p*-values < 0.05). The complete statistical results are reported in Table 2. Forced-choice questions attained similar results (Figure 3B). The majority of the participants preferred condition TG with every object of the experiment, perceiving it as faster and easier than condition UG.

**Table 3:** Questionnaire and internal consistency of each set of questions.

**Questionnaire**
**Q1. Robot Contribution** α = 0.822
1.1. I had to carry the weight to make the human-robot team better. (reverse scale)
1.2. The robot contributed equally to the team performance.
1.3. The robot's performance was an important contribution to the success of the team.
**Q2. Working alliance Index (WAI)—goal subscale** α = 0.859
2.1. The robot minded what my goal was.
2.2. The robot did not take into account what I was going to accomplish (reverse scale).
2.3. The robot and I were working toward the same goals.
**Q3. Easy grasping** α = 0.913
3.1. The robot left enough clear space on the object to allows me to easily grasp the object
3.2. The robot gripper obstructed me from grasping the object (reverse scale).
3.3. I could easily receive the object from the robot.
**Q4. Direct use of the object** α = 0.959
4.1. After having received the object from the robot, I needed to re-adjust the object position inside my hand to complete the task. (reverse scale)
4.2. The robot handed over the object so that I could use the object right away for the following task.
4.3. The robot presented the object to make it faster for me using the object for the following task.
**Q5. Forced-Choice Questions (answer with First condition or Second condition)** α = 0.899
5.1. Which program were you the fastest with?
5.2. Which program was the easiest?
5.3. Which program did you prefer?

*Questionnaire administered to the participants after performing both conditions (UG and TG) with each object. Each question of the first four sets were rated using a 7-point Likert scale (1 strongly disagree; 7 strongly agree), while in the forced questions (set Q5) participants were asked to choose between one of the two conditions. The internal consistency of each set of the questionnaire is reported using Cronbach's alpha (α)*.

## 4. Discussion

The choice of a grasp is a complicated process said to depend on multiple factors (Napier, 1956; Kamakura et al., 1980; Cutkosky, 1989; Iberall, 1997; Lukos et al., 2007; Feix et al., 2014b, 2016). The task to perform plays an important role in such choice (Ansuini et al., 2006, 2008; Feix et al., 2014a; Vergara et al., 2014; Hjelm et al., 2015; Detry et al., 2017; Cini et al., 2019). The robotics community proposed grasping strategies (Adjigble et al., 2018; Morrison et al., 2020) whose success is defined using traditional metrics, such as stability (Bicchi and Kumar, 2000), and speed (Mahler et al., 2018). However, task-oriented grasping has gained momentum recently, especially thanks to improved techniques in vision and learning (Do et al., 2018; Cavalli et al., 2019), and metrics shaped by the task (Ortenzi et al., 2019). Choosing a grasp is important when a robot has to directly perform a task; and, arguably, even more important when the robot has to interact and collaborate with another agent. Previous studies have investigated different grasping strategies to enable robots to fluently hand over an object, but without including any following activity in their protocol (Cakmak et al., 2011a; Aleotti et al., 2014; Chan et al., 2019). However, an object handover is usually performed by a dyad to allow the receiver to accomplish a further task in the shortest possible time. Therefore, the grasping strategy used by the passer to present the object, may affect both the receiver's performance and perception along the entire task (not only during the handover). Our work addresses this open issue carrying out a study with robotic-naïve participants (i.e., not accustomed to working with robots) who had to receive five different objects from a robot and perform a specific task afterwards. We asked the subjects to repeat the same task under two different conditions (UG and TG) and to perform the task as fast as possible. Condition UG leverages only on canonical grasp stability considerations. Differently, TG accounts for the purposive action of the handover and orientates the object's grasping affordance toward the user while leaving it encumbered. Our results show that the robotic grasping strategy does not affect the receiver's reaching time. However, a task-oriented grasp reduces the number of object manipulations performed by the receiver after the handover, increasing the efficiency of the subsequent task and improving the perception of the interaction. The passer's grasping strategy is determinant especially for those objects whose affordances and use introduce strong constraints in the manipulative actions of the receiver.

### 4.1. Human Receivers Prefer a Fast and Provisional Grasp

The time spent by the receivers to reach the robotic gripper (*T*_*reach*_) was not affected by the robot's grasping strategy, i.e., how the robot obstructs and orientates the object toward the receiver (Figure 2A). So far, the effect of the robot's grasping strategy on the receiver's reaching movement is yet to be determined, since previous studies present contrasting results. In a preliminary analysis, Aleotti et al. (2014) suggested that the time spent by the receiver to reach for the object after the robot has stopped, decreases when the object is presented with its affordance clear and oriented toward the receiver. However, two main differences occur when comparing that study with our work. First, participants in Aleotti et al. (2014) had the only goal to receive an object from the robot, with no subsequent task to perform. Perhaps more importantly, in this previous study, participants were not stressed by or concerned about their performances (contrary to our protocol). Authors did not give any indication on performing the task as fast as possible and participants were allowed to interact with the experimenter to decide when to grasp the object. Differently, results achieved by Cakmak et al. (2011a) are in line with our results, as they observed that different grasping strategies of the robotic passer did not influence the time spent by the receiver to reach for the object. Similar to our work, but in contrast to the study of Aleotti et al. (2014), the experiment carried out by Cakmak et al. requested that participants performed the task as quickly as possible. However, even in that case, there was no further action to perform with the object after the handover, i.e., obtaining the object during the handover represented the only task to perform for the receiver. The comparison of our results and protocol with those of Cakmak et al. (2011a) and Aleotti et al. (2014), suggests that, under given time constraints, participants prioritize a quick, albeit provisional, grasp to obtain the control of the object. Such behavior is in agreement with previous research in neuroscience showing that the motor control strategy used by humans breaks manipulative tasks into a series of action-phases delimited by sub-goals (Randall Flanagan et al., 2006; Johansson and Flanagan, 2009). Then, the brain must choose the optimal action-phase controller that satisfies some efficiency criterion to achieve the sub-goals, such as a minimization of energy consumption, execution time, and motion uncertainties (Engelbrecht, 2001). Optimal control models result in maximizing the smoothness of the hand trajectory (Flash and Hogan, 1985) and the joints' torque commands (Uno et al., 1989) avoiding large and complex movements (Wolpert and Ghahramani, 2000). Thereby, it is likely that during the reaching phase—that is the first sub-action in the handover—the primary goal of the receiver is to quickly apprehend the object, and the most comfortable and smooth approaching movement is chosen. Consequently, if the robot presents the object in a non-suitable configuration for the partner to perform the subsequent task, receivers prefer a provisional grasp, rather than carrying on a longer and non-efficient movement to compensate the non-ideal object's position and/or orientation. Although provisional grasps come handy for the receiver, they usually do not allow to perform the subsequent operations. Receivers must further manipulate the object to obtain a comfortable and usable grasp. In line with this hypothesis, our results show that when the robot's grasping strategy did not account for the receiver's subsequent task (UG condition), participants resorted to in-hand or bimanual re-adjustments soon after the handover in more than 70% of the trials (Figures 2C,D). The use of manipulative adjustments dropped to 21% when the robot passed the object in an appropriate way (TG condition). Only the drill led to a number of in-hand manipulations higher than 20% in TG. We believe that this was due to a non-ergonomic handover location. For some participants the handle of the drill was located too high and once grasped, they needed to shift it inside their hand. However, no-adjustments in TG (53%) were considerably more frequent than in UG (7%) also in this case. A reduced difference in the number of no-adjustments in UG and TG was observed instead with screwdriver and mustard bottle. The recurrent use of a provisional grasp and subsequent re-manipulations observed in UG, negatively affected the efficiency of the receiver during the subsequent task. We observed that for all the objects, but the mustard bottle, the task completion time was significantly higher in UG than in TG (Figure 2B). Results in terms of the required time suggest that the robot grasping strategy has a strong impact on the efficiency of the receiver's subsequent task, but not in the receiver's reaching phase. However, the dissimilar results on the object manipulations obtained with the mustard bottle and the screwdriver suggest that effects of the passer's grasping strategy may not be independent from the object that has to be passed and then used. This issue will be discussed in detail in a subsequent paragraph.

### 4.2. The Perceived Quality of the Interaction Improves With a Clear Availability of the Grasping Affordance

Previous studies showed that human's perceptions about their interaction with robots may not directly reflect the objective measures of task efficiency (Hoffman and Breazeal, 2007; Huber et al., 2008a). This implies that while metrics, such as task completion time or object re-adjustments can be used to objectively evaluate the efficiency of a collaborative task, they cannot be used to assess the receiver's perceptions and preference (Cakmak et al., 2011b; Hoffman, 2019). Thus, in this work, a questionnaire (Table 3) was used to assess whether the robot's grasping strategy affects how humans perceive: (i) the contribution of the robot to the team action and goal (Q1 and Q2); (ii) the easiness and speed of execution of the collaborative task (Q3 and Q4); (iii) the preference among the two robot behaviors (Q5). The statistical analysis showed that the effect of the passer's grasping strategy on the ratings of Q1 and Q2 was significant for all the objects but the mustard bottle. The ratings of Q3 and Q4 significantly changed across conditions for all the objects without exception (Figure 3A). In addition, the ratings of the forced-choice questions confirmed that, regardless of the object, participants strongly preferred TG, perceiving it as easier and faster than UG (Figure 3B). These outcomes suggest that if the robot presents an object considering the receiver's needs, humans are more inclined to perceive it as a collaborative partner committed to the team goal rather than as an inactive agent or tool (Horvath and Greenberg, 1989; Hoffman, 2019). In addition, when the receiver has to perform a subsequent task as fast as possible and the object is offered with grasping affordance obstructed or/and not properly oriented, the grasping action and the following task are perceived more difficult and slower even when the task completion time did not actually worsen (as observed with the mustard bottle, Figure 2B). Thus, it is likely that the recurrent use of a provisional grasp and subsequent re-manipulations observed in UG, not only increased the receivers' physical effort but also their cognitive fatigue. The latter may have led to a degradation of the human perception of the interaction even when the re-manipulations were easy and fast and did not affect the efficiency of the task. Cognitive and physical fatigue during human-robot collaboration is a crucial issue especially in industrial settings (Bascetta and Ferretti, 2019; Nikolakis et al., 2019; Peternel et al., 2019). In such conditions, workers repeat the same action for long periods of time and even small compensatory movements could produce an excessive level of physical and cognitive effort that may result in an increase of human mistakes or physical disorders (Peternel et al., 2019). Thereby, introducing robots that adjust their behavior according to their partner's needs could improve not only the efficiency but also the well-being of workers and thus, the efficiency of the entire production process.

### 4.3. Hand-Object Interface and Tool-Use Action Influence the Effect of the Passer's Grasping Strategy

The concept of object affordances was coined by Gibson (1977, 1979). Since then, this notion has acquired different and sometimes ambiguous meanings (Ellis and Tucker, 2000; Norman, 2002; Young, 2006; Borghi and Riggio, 2015). Recently, Osiurak et al. (2017) offered a clarified definition that, similar to Gibson, describes affordances as object's properties suggesting an action possibility. Even if affordances are physical properties of a tool, they also depend on the individual's action capabilities and therefore, they can vary among species and individuals of the same species. The mechanical action of using an object involves two different interfaces (Osiurak and Badets, 2016; Osiurak et al., 2017): (i) the physical interface between the human hand and the object's affordance (hand-object interface), and (ii) the interaction between the object and the environment needed to realize the object's usage (object-use interface). For the sake of completeness, in this work the term object-use interface will not imply only the physical characteristics that the object and the environment must have in order to actualize the object's usage, as in Osiurak and Badets (2016), but it will include also any reduction of the number of all possible object-environment interactions due to the limited human's body capabilities. The five objects used in this experiment can be seen as representative of five possible combinations of different hand-affordance and object-use interfaces (Table 4). The mustard bottle represents a class of objects whose interfaces are little restrictive. The affordance of the mustard bottle involved in the interaction with the human hand is its whole body (wide surface) and both its minor dimensions are easily graspable by the human hand. These physical characteristics allow the human fingers to easily wrap the affordance with unlimited possible orientations around its major axis. Similarly, its object-use interface allows unlimited rotations around the bottle's major axis with the only restriction to leave the top of the bottle free (to allow pouring). The screwdriver has similar characteristics, but its usage implies an additional requirement: the tip of the object must be precisely centered on the screw head. The drill represents a class of objects with intermediate constraints. Even though its grasping affordance—the handle—is wide, it has been designed to be grasped with a preferential orientation. In addition, similarly to the screwdriver, the drill requires the tip to be accurately positioned in a fixed location of the work-piece, and it can be ideally used with a multitude of postures around the axis perpendicular to the handle's major dimension. However, the unlimited number of orientations suitable to use the object is reduced by the limited ergonomic postures that the human arm can assume during the tool-use action. A similar restriction occurs also when a human being grasps a mug by its handle (to pour something in it) or scissors (to cut). In addition, the tool-use interface requires to center the clear cavity of the mug with the spout of the jug and to position the scissors' blades around the target to cut, respectively. However, these positioning constraints require less accuracy, and thus are less limiting than those required by tools as screwdriver or drill. On the contrary, the hand-affordance interfaces of the mug and of the scissors are quite restrictive as they entail a precise control of the hand to insert the fingers in the narrow holes. Examining all our results in light of the constraints induced by each object, another interesting discussion point emerges: the effect of the passer's grasping strategy on the efficiency and perception of the receiver is affected by the hand-affordance and tool-use interfaces of the objects involved in the interaction (Table 4). Our outcomes show that with objects, such as the mustard bottle and screwdriver, whose interfaces with the hand and the environment are less restrictive, TG led to limited improvements with respect to UG. In particular, even if the body of the mustard bottle was presented with an adverse orientation and the handle of the screwdriver was substantially obstructed in UG, participants were still able to receive and use these tools without adjustments in more than 50% of cases (Figure 2C). Thus, TG produced only a slight increase of no-adjustments with respect to UG, which, in the case of the mustard bottle, was not enough to obtain a significant reduction of the task completion time (Figure 2B). As for the mustard bottle, we also did not find a significant effect of the passer's grasping strategy on the receiver's perception of the robot as an ally (Q1 and Q2 in Figure 3A). These results suggest that when the constraints required by the hand-affordance interface and tool-use interface are very light, the facilitations provided by a more complex robotic grasping strategy, that accounts for the needs of the receiver, may not be enough for the robotic passer to be perceived as more supportive or to significantly improve the efficiency of the collaboration. In contrast, when the actualization of the object's usage has more constraints (as with the drill) or the affordances require more precise hand's movements to be grasped (as with mug and scissors), the robot grasping strategy becomes determinant for both an efficient completion of the task and for the receiver's perceptions of the interaction. Our results show that the occlusion of the mug's handle (even if maintained in its correct orientation) and the adverse rotation of the drill's hilt (even if completely unobstructed) induce a degradation of both the task efficiency and the receiver's perception.

**Table 4:** Hand-affordance interface and object-use interface of each test-objects and relative effect of TG.

**Object: hand-object/tool-use interface (Score)**	**Hand-object interface (Sub-score)**	**Tool-use interface (Sub-score)**	**UG impediments**	**Positive effects of TG w.r.t. UG**	
				**Efficiency**	**Perception**
**Mustard bottle**: Body/Mouth (**Score = 0**)	(**Sub-score = 0**) **+** The affordance is wide, and the hand can easily wrap it (**0**) **+** Unlimited grasping possibilities rotating the hand around the object major axis (**0**)	(**Sub-score = 0**) **+** The mouth must be free (**0**) **+** Unlimited orientations along the major axis of the object are suitable for the task (**0**)	**Occlusion**: no occlusion of the affordance. **Orientation**: adverse orientation of the affordance.	No improvements in task completion time. Moderate increase of no- adjustments.	No improvements in perception of robot as an ally. Improvement in perception of easiness and speed of the task execution.
**Screwdriver**: Handle/Tip (**Score =** **−1**)	(**Sub-score = 0**) **+** The affordance is wide, and the hand can easily wrap it (**0**) **+** Unlimited grasping possibilities rotating the hand around the object major axis (**0**)	(**Sub-score =** **−1**) **+** The tip must be free (**0**) **+** The tip must be centered on the screw (**−1**) **+** Unlimited orientations along the major axis of the object are suitable for the task (**0**)	**Occlusion**: partial occlusion of the affordance. **Orientation**: correct.	Improvement in task completion time. Negligible increase in the number of no-adjustments.	Improvements in perception of robot as an ally. Improvement in perception of easiness and speed of the task execution.
**Drill**: Handle/Drill bit (**Score =** **−2.5**)	(**Sub-score =** **−1**) **+** The affordance is wide, and the hand can easily wrap it (**0**) **+** The handle is designed to be grasped with a preferential orientation (**−1**)	(**Sub-score =** **−1.5**) **+** The tip must be free (**0**) **+** The tip must be aligned (**−1**) **+** Reduction of the orientations along one axis due to the limited human arm postures (**−0.5**)	**Occlusion**: None. **Orientation**: adverse orientation of the affordance.	Improvement in task completion time. Increase of the number of no-adjustments.	Improvements in perception of robot as an ally. Improvement in perception of easiness and speed of the task execution.
**Mug**: Handle with single hole/Mouth (**Score =** **−3**)	(**Sub-score =** **−2**) **+** The affordance size is reduced and requires a precise control of the hand to insert the fingers (**-2**)	(**Sub-score =** **−1**) **+** The mouth must be free (**0**) **+** The mouth must be aligned with the jug (**−0.5**) **+** Reduction of the orientations along one axis due to the limited human arm postures (**−0.5**)	**Occlusion**: occlusion of the affordance. **Orientation**: correct.	Improvement of task completion time. Increase of the number of no-adjustments.	Improvement in perception of robot as an ally. Improvement in perception of easiness and speed of the task execution.
**Scissors**: Handle with two finger-holes/Blades (**Score =** **−3**)	(**Sub-score =** **−2**) **+** The affordance size is reduced and requires a very precise control of the hand to insert thumb and another finger in the two holes of the handle (**−2**)	(**Sub-score =** **−1**) **+** The blades must be free (**0**) **+** The blade must be positioned (**−0.5**) **+** The unlimited orientations along the major axis that are suitable for the task are reduced by the human arm limited posture (**−0.5**)	**Occlusion**: Partial occlusion of the affordance. **Orientation**: adverse orientation of the affordance.	Improvement of task completion time. Increase of the number of no-adjustments.	Improvement in perception of robot as an ally. Improvement in perception of easiness and speed of the task execution.

*Constraints induced by the hand-affordance interface and the tool-use interface for each of the five test-objects. Constraints were compared using a negative scale, where the more restrictive a constraint is, the lower its score (in bold). Since the constraints of the mustard bottle were the less restrictive, they were used as reference (0). We also report the impediments introduced in UG and the positive effects of TG. Scores and sub-scores are reported in bold for illustrative purpose only*.

## 5. Conclusion

Overall, our results suggest that human receivers prefer a fast and provisional grasp during the handover when asked to execute a task as fast as possible. Then re-adjustments are made if the provisional grasp does not allow to ergonomically and efficiently perform the task with the object. To answer the original questions about the impact of the grasping strategy of a robotic passer, our work suggests that while there is no difference in terms of reaching time (to obtain the object from the robot passer), the performances of human receivers are generally improved with a task-oriented strategy adopted by the robot. Moreover, the perceived quality of the interaction improves when the grasping affordance of the object is available to the receivers, who then appreciate the robot as an ally. In this perspective, hand-object interface and tool-use action must be considered by the robot's grasping strategy. The more restrictive the constraints for the use of the object are, the more influential the grasp strategy of the passer becomes.

Although the experimental protocol used in this study was developed to test the effect of the grasping choice of the robotic passer on the performance and subjective perception of the interaction of the receiver, we believe that this protocol can be exploited as is or adapted for testing and benchmarking other aspects connected to the effects of different grasping strategies by the passer.

Future work includes the implementation of the studied policies with a fully automated grasping pipeline, which would enable us to test their beneficial effect in long-lasting Human Robot Interaction. Furthermore, our results reflect a general standpoint of the robot-human handover; however, we are considering to run a user-study in a specialized work environment, such as a factory, where skilled workers interact with the robotic passer, the used tools present more distinctive functional parts, and the time constraints are more stringent.

## Data Availability Statement

The datasets generated for this study are available on request to the corresponding author.

## Ethics Statement

The studies involving human participants were reviewed and approved by the Science, Technology, Engineering and Mathematics Ethical Review Committee at the University of Birmingham, UK. The patients/participants provided their written informed consent to participate in this study. Written informed consent was obtained from the individual(s) for the publication of any potentially identifiable images or data included in this article.

## Author Contributions

VO and MC initiated this work, oversaw, and advised the research. MC, FC, and VO designed the experiments. VO, NM, and TP built the experimental setup. VO, TP, FC, and MC performed the experiments. RS and MC provided the financial support. All authors contributed to the article and approved the submitted version.

## Conflict of Interest

The authors declare that the research was conducted in the absence of any commercial or financial relationships that could be construed as a potential conflict of interest.

## Supplementary Material

Video S1 The video shows a mock-up of the experimental setup and protocol. In particular examples of both the experimental conditions UG and TG with different test-objects are displayed.
